# Impact of dietary rumen degradable protein level on growth performance, rumen fermentation, and nitrogen utilization in growing Hu sheep

**DOI:** 10.1093/tas/txag011

**Published:** 2026-03-07

**Authors:** Yu Zang, Jun Zhu, Xinhuang Zhong, Asmita Thapa, Airong Zhu, Peihua You, Mengzhi Wang

**Affiliations:** College of Animal Science and Technology, Yangzhou University, Yangzhou, Jiangsu, 225009, China; State Key Laboratory of Sheep Genetic Improvement and Healthy Production, Xinjiang Academy of Agricultural Reclamation Sciences, Shihezi, Xinjiang, 832000, China; College of Animal Science and Technology, Yangzhou University, Yangzhou, Jiangsu, 225009, China; College of Animal Science and Technology, Yangzhou University, Yangzhou, Jiangsu, 225009, China; College of Animal Science and Technology, Yangzhou University, Yangzhou, Jiangsu, 225009, China; Jiangsu AgriPortal Co. Ltd, Nanjing, Jiangsu, 211803, China; Jiangsu AgriPortal Co. Ltd, Nanjing, Jiangsu, 211803, China; College of Animal Science and Technology, Yangzhou University, Yangzhou, Jiangsu, 225009, China

**Keywords:** digestibility, fermentation, growth, rumen degradable protein, sheep

## Abstract

It is critical to supply adequate rumen degradable protein (**RDP**) to maintain ruminal fermentation of carbohydrates and microbial protein synthesis in sheep. Our objective was to investigate the effect of dietary RDP content on growth performance, rumen fermentation, and nitrogen utilization in growing Hu sheep. The study was conducted for 11 wk (i.e., 2-wk adaptation period and 9-wk experimental period) with 44 3-month-old intact male Hu sheep averaging body weight (**BW**) of 22.7 ± 3.32 kg at the beginning of the trial. Following the adaptation period, sheep were blocked by BW and randomly assigned to 1 of 4 experimental diets. Dietary RDP concentrations of 11.0%, 10.0%, 9.0%, and 8.0% were achieved by replacing solvent soybean meal and wheat middlings with coarsely cracked corn and extruded soybean meal, and experimental diets were fed as pelleted total mixed rations. Treatment had no effect on BW, average daily gain, dry matter intake, feed conversion ratio, or income over feed cost. When decreasing dietary RDP, body height decreased (*P* *=* 0.017) linearly, chest depth tended (*P* *=* 0.079) to reduce linearly, and chest width responded (*P* *=* 0.008) quadratically. There were (*P* *≤* 0.018) quadratic effects of decreasing RDP concentration for ruminal concentrations of total volatile fatty acids (**VFA**), acetate, butyrate, and branched-chain VFA (i.e., isobutyrate, valerate, and isovalerate), with maximal values at 10.0% RDP. Apparent total-tract digestibilities of crude protein and acid detergent fiber decreased (*P* *≤* 0.001) and that of starch increased (*P* *=* 0.003) linearly as dietary RDP concentration decreased from 11.0% to 8.0%. Decreasing RDP tended (*P* *=* 0.051) to have a quadratic effect on NDF digestibility. Urinary urea-nitrogen and total-nitrogen excretion showed (*P* *<* 0.001) linear decreases when dietary RDP concentration declined. There was (*P* *=* 0.027) a cubic effect of decreasing RDP on fecal nitrogen output. The ratio of retained nitrogen to nitrogen intake was maximized at 10.0% RDP (quadratic effect, *P* *=* 0.016). In summary, even though reducing dietary RDP level from 11.0% to 8.0% did not modify growth performance and linearly reduced urinary N output, ruminal fermentation, apparent total-tract digestibility of NDF, and nitrogen utilization were optimal when feeding the 10.0% RDP diet.

## Teaser

Currently, there are no recommendations for rumen degradable protein in growing Hu sheep. This study has established an optimal dietary level of 10% of ration to enhance rumen fermentation, neutral detergent fiber digestibility, and nitrogen utilization.

## Introduction

The poor efficiency of nitrogen (**N**) utilization in both monogastric and ruminant animals has led to elevated demand for protein feeds and exacerbated environmental N pollution (Castillo et al. 2000; Jones et al. 2014). China, as a top global player in the livestock sector, has a substantial demand for protein feeds such as soybean meal. Therefore, in 2023, the Ministry of Agriculture and Rural Affairs of China released the “Three-Year Action Plan for Reducing Soybean Meal in Feed”, calling for more investigations into low-protein rations, alternative protein feedstuffs, and high-protein forages (MOA 2023). In ruminants, dietary crude protein (CP) can be categorized into rumen degradable protein (RDP) and rumen undegradable protein (RUP), with RDP utilized for microbial protein synthesis, and microbial protein and RUP serving as sources of metabolizable protein in the small intestine (NASEM 2016, 2021). Considering the distinct features and roles of RDP and RUP, it is critical to separately investigate them in order to develop low-protein diets without compromising production performance in sheep.

Feeding sufficient RDP can optimize microbial protein synthesis and ruminal carbohydrate fermentation (Lee et al. 2012; NASEM 2021), thereby contributing amino acids (**AA**) and energy to ruminants. Nevertheless, when excess RDP is provided, RDP not incorporated into microbial protein is deaminated and converted to ammonia N, which is ultimately being synthesized into urea in the liver (Reynolds 1992; Bach et al. 2005). This process both decreases N utilization and demands extra energy; thus, determining an ideal level of dietary RDP is of significance for efficient N utilization and optimum ruminal fermentation. Ross et al. (2024) examined 3 dietary RDP levels (i.e., 8, 9, and 10% of ration dry matter [DM]) in finishing beef steers and reported that retained protein showed a quadratic pattern, achieving its maximum value at 9% RDP. There have also been several dairy studies investigating different dietary levels of RDP while RUP was kept constant (Reynal and Broderick 2005; Kalscheur et al. 2006). Specifically, Reynal and Broderick (2005) formulated diets with different concentrations of RDP (i.e., 13.2%, 12.3%, 11.7%, and 10.6%) and reported that dry matter intake (**DMI**) and milk true protein yield were maximized at 12.3% and 11.7% RDP. Moreover, yields of milk and milk components showed linear decreases as dietary RDP decreased from 11.0% to 6.8% of ration (Kalscheur et al. 2006). Based on these prior studies (Reynal and Broderick 2005; Kalscheur et al. 2006), the committee on Nutrient Requirements of Dairy Cattle recommended a range of 10% to 12% of RDP in dairy rations (NASEM 2021).

To the best of our knowledge, there is scarce information on appropriate RDP level for growing Hu sheep. The objective of our study was to evaluate the impact of dietary RDP concentration on growth performance, ruminal fermentation, and N utilization in growing Hu sheep. We hypothesized that the efficiency of N utilization would be enhanced with decreasing RDP levels, but growth performance, rumen fermentation, and nutrient digestibility would be negatively impacted when supplying RDP below the requirement. We anticipate that our findings will determine an optimal dietary RDP concentration for growing Hu sheep to enhance N use efficiency and reduce manure N excretion while maintaining growth performance and rumen fermentation.

## Materials and methods

All study procedures were approved by Yangzhou University Animal Care and Use Committee. The study was conducted at the Sheep Research Center of Yangzhou University (Yangzhou, Jiangsu) from April to June 2024.

### Animal management and experimental design

Forty-four 3-month-old intact male Hu sheep averaging (mean ± SD) 22.7 ± 3.32 kg of body weight (**BW**) were enrolled at the beginning of the experiment. Sheep were housed in individual stalls with feed tubs for individualized feed intake and nipple drinkers for ad libitum access to water. Sheep were fed twice daily at 0830 and 1630 h, and refusals were collected daily before the morning feeding. Feed offered was adjusted daily to allow for 5% to 10% refusals.

The experiment lasted 11 wk, including a 2-wk adaptation period and a 9-wk experimental period. During the adaptation period, all the animals were fed a commercial pelleted total mixed ration (**TMR**) produced and delivered by Jiangsu AgriPortal Co. Ltd (Nanjing, Jiangsu). The commercial ration contained (% of DM) 15.6% CP, 32.1% starch, 30.8% neutral detergent fiber (**NDF**), 17.4% acid detergent fiber (**ADF**), 2.82% ether extract, and 8.15% ash. Following the adaptation period, sheep were blocked by BW and, within each block, assigned randomly to 1 of 4 experimental diets. Diets were fed as pelleted TMR with a forage to concentrate ratio of 48:52. The forage included rice straw and wheat straw, and the concentrate contained coarsely cracked corn, solvent soybean meal, extruded soybean meal, rice bran, wheat middlings, urea, and the mixture of mineral and vitamins. Solvent soybean meal and wheat middlings were replaced with coarsely cracked corn and extruded soybean meal to achieve dietary RDP levels of 11.0%, 10.0%, 9.0%, and 8.0%, while dietary RUP was kept relatively constant (from 6.25% to 5.25%). Dietary RDP levels were chose based on previous dairy and beef studies investigating varying RDP content from 6.8% to 13.2% (Reynolds 2005; Kalscheur et al. 2006; Ross et al. 2024). Diets were formulated to meet the nutrient requirements, except MP, of a growing sheep averaging 35 kg of BW, consuming 1.75 kg/d of DM, and gain 300 g/d of BW, using a formulation software AMTS Nutrition with Small Ruminants Module based on NRC (2007).

### Samplings and measurements

Body weight was recorded at the beginning and the end of the experimental period to monitor changes in BW and calculate average daily gain (**ADG**). Feed offered and refusals were weighed daily throughout the experiment to calculate feed intake. Income over feed costs was calculated as the revenue minus the expense of pelleted total mixed ration. Samples of pelleted TMR and refusals were collected for 3 consecutive days every 3 wk and were dried at 55°C for 48 h in a forced-air oven (101-4BS, Shangcheng Instrument Manufacturing, Shaoxing, China) for DM determination and DMI calculation. During the final week of the experimental period, body measurements (i.e., wither height, body height, rump height, body length, chest depth, chest width, chest girth) were recorded following the procedure reported by Esquivelzeta et al. (2011).

Samples of rice straw, wheat straw, coarsely cracked corn, solvent soybean meal, extruded soybean meal, rice bran, wheat middlings, urea, and the premix of mineral and vitamins were collected once before the pelleted TMR were produced at Jiangsu AgriPortal Co. Ltd (Nantong, Jiangsu). Then, pelleted TMR were sent to the Sheep Research Center of Yangzhou University (Yangzhou, Jiangsu) to be used throughout the experiment. Samples of individual ingredients were shipped to Cumberland Valley Analytical Services (Tangshan, Hebei) and analyzed for DM, CP, soluble protein, neutral detergent insoluble CP (**NDICP**), acid detergent insoluble CP (**ADICP**), NDF, ADF, acid detergent lignin (**ADL**), starch, water soluble carbohydrates (**WSC**), ether extract, ash, and minerals (i.e., Ca, P, Mg, K, S, Na, Cl, Fe, Mn, Zn, and Cu) following the procedures previously mentioned by Zang et al. (2024). After the trial, growth performance of sheep and analyzed nutrient composition of ingredients were entered into the AMTS Nutrition with Small Ruminants software for dietary evaluation (Rius et al. 2010; Zang et al. 2021).

On d 77 of the experimental period, rumen fluid was collected approximately 4 h after the morning feeding, using the esophageal tubing technique. The initial 50 mL of rumen fluid was discarded to avoid saliva contamination, and another 50 mL of ruminal fluid was then collected into a 100 mL container. Following collection, the pH was immediately measured using a pH meter (PH-100A, Lichen-Bangxi Instrument Technology, Shanghai, China) and 10 mL of sample was added into a 15-mL centrifuge tube with 200 µL of 50% sulfuric acid (vol/vol) for the analysis of volatile fatty acids (**VFA**). Following a 10-fold dilution of the rumen fluid, 100 μL of supernatant was added to 200 μL of acetonitrile containing [2H9]-pentanoic acid and [2H11]-hexanoic acid as internal standards, being vortexed for 1 min and extracted by sonication in an ice-water bath for 10 min. The extract was centrifuged at 17,800 × g for 10 min at 4°C, and the supernatant was collected for further processing. 80 μL of supernatant was taken, 40 μL of 200 mM 3-NPH (50% acetonitrile, vol/vol) and 40 μL of 120 mM EDC-6% pyridine (50% acetonitrile, vol/vol) were added, reacted at 40°C for 30 min, cooled on ice for 1 min, and then stored at −80°C until analysis. The analysis was performed using an ultra-high performance liquid chromatography-mass spectrometer [UHPLC-MS; QTRAP ®6500+ (AB Sciex, MA, USA)]. Chromatographic separation was carried out on a Waters ACQUITY UPLC BEH C18 precolumn (1.7 μm, 2.1 × 100 mm). The mobile phase contained solvent A (i.e., 0.1% acetic acid, vol/vol) and solvent B (acetonitrile-to-methanol, 2:1, vol/vol). The gradient elution procedures were as follows: 0 min A/B (75:25, vol/vol), 2 min A/B (75:25, vol/vol), 11 min A/B (45:55, vol/vol), 12 min A/B (75:25, vol/vol), and 13 min A/B (75:25, vol/vol).

During the final 3 d of the experimental period, customized cloth bags were used to collect fecal samples at 8 time points as follows: 0800 and 1700 h (d 78), 0200, 1100, and 2000 h (d 79), and 0500, 1400, and 2300 h (d 80). During each sampling, 50 g of fecal samples were taken and transferred into 1-L plastic bags to obtain a composite sample (wet weight) by sheep, and stored at −20°C until further processing. Samples were thawed at room temperature, placed in aluminum trays, dried for 72 h at 55°C in a forced-air oven, and ground to pass through a 1-mm screen (Wiley mill; A. H. Thomas Co.). Dried fecal samples were shipped to Cumberland Valley Analytical Services for analyses of DM, CP, NDF, ADF, ADL, starch, ether extract, and ash as analyzed for individual ingredients. Acid detergent lignin was used as the internal marker to estimate fecal output of DM and apparent total-tract digestibility of nutrients (Krysl et al. 1988).

Spot samples of urine (∼15 mL) were collected concurrently with feces by using a customized urine collection device. For each sampling, 1 mL of urine (total = 8 mL over the sampling period) was pipetted into 15-mL tubes containing 200 μL of 50% sulfuric acid (vol/vol) and kept refrigerated at 4°C until the sampling was completed. Then, composite samples of urine were stored at −20°C before analyses of nitrogenous metabolites. Samples were thawed inside a refrigerator (4°C) and analyzed for creatinine (assay kit no. 500701; Cayman Chemical Co.) using a chromate microplate reader set at a wavelength of 492 nm (Awareness Technology Inc.), urea-N (Stanbio Urea Nitrogen Kit 580; Stanbio Laboratory Inc.) with a UV/visible spectrophotometer (Beckman Coulter Inc.) set at a wavelength of 520 nm, and total-N [micro-Kjeldahl analysis (AOAC 1990); Cumberland Valley Analytical Services]. Daily urinary output was calculated assuming a constant creatinine excretion rate of 15.0 mg/kg of BW (Pereira et al. 2022). Body weight measured on d 78 was used to estimate urinary volume. Urinary excretion of urea nitrogen and total nitrogen was calculated by multiplying their concentration in urine by estimated urine volume. The amount of retained nitrogen was determined by subtracting nitrogen excreted in feces and urine from total nitrogen intake.

### Statistical analysis

Data were analyzed as a randomized block design using the MIXED procedure of SAS (SAS version 9.4; SAS Institute Inc., Cary, NC) ­according to the following model:


$$Y_{\text{ijk}}=\mu +B_{i}+T_{j}+e_{\text{ij}}$$

where, Y_ijk_ = dependent variable, μ = overall mean, B_i_ = random effect of block (i = 1 to 11), T_k_ = fixed effect of treatment (k = 1 to 4), and e_ijk_ = residual error. Normality of the residuals was checked with normal probability and box plots and homogeneity of variances with plots of residual versus predicted values. Data were considered as outliers and removed from analysis when Studentized residuals were >3.0 or <−3.0. Preplanned polynomial orthogonal contrasts were used to test linear, quadratic and cubic effects in response to incremental levels of dietary RDP. Mean separation was performed using the Tukey test. All data were ­reported as least square means ± SEM. Significance was declared at *P* *≤* 0.05 and trends at 0.05 < *P* *≤* 0.10.

## Results

### Diet composition

Nutrient composition of individual ingredients used in experimental diets is shown in Table 1. Ingredient and chemical composition of experimental diets are represented in Table 2. Extruded soybean meal and coarsely cracked corn were used to replace soybean meal and wheat middlings to achieve decreasing ­levels of RDP (i.e., 10.9% to 8.09%), while dietary RUP decreased slightly (i.e., 6.25% to 5.25%). Experimental diets had similar concentrations of NDF (40.5% ± 0.05%), ADF (25.7% ± 0.26%), WSC (6.93% ± 0.62%), and ether extract (2.90% ± 0.02%), while dietary starch content changed from 23.3% to 28.6% as dietary RDP content decreased. Notably, NRC (2007)-estimated supplies of metabolizable energy (**ME**) and MP, expressed as % of the requirements, ranged from 170% to 185% and 179% to 169%, respectively.

**Table 1:** Nutrient composition of ingredients used in experimental diets (% of DM, unless otherwise noted).

Item	Rice straw	Wheat straw	Coarsely cracked corn	**Solvent SBM** ^a^	Extruded SBM	Rice bran	Wheat middlings	**Minerals & Vitamins** ^b^
**DM, % of as fed**	89.2	89.5	88.5	87.8	87.9	88.1	87.7	92.6
**CP**	5.00	3.30	8.40	48.8	48.5	14.5	19.4	62.5
**SP^c^, % of CP**	26.6	29.4	25.9	27.1	9.50	27.4	49.1	97.1
**ADICP**	0.54	0.60	0.21	0.31	0.49	0.29	0.20	0.21
**NDICP**	1.71	1.03	0.41	1.16	7.72	0.78	1.17	0.28
**NDF**	68.9	79.0	7.90	12.3	20.6	19.4	30.6	21.6
**ADF**	45.8	51.3	2.50	9.30	8.00	12.5	8.60	13.8
**ADL**	4.45	6.46	0.99	0.33	0.13	3.04	1.80	4.39
**WSC**	5.80	4.10	4.30	21.1	19.5	10.6	13.5	1.40
**Starch**	3.10	0.90	73.8	6.90	6.00	34.5	28.3	0.70
**Ether extract**	2.12	1.00	4.29	2.07	0.89	10.6	5.16	1.38
**Ash**	4.45	10.0	1.44	6.98	7.23	7.69	4.47	46.76
**Ca**	0.53	0.28	0.03	0.29	0.35	0.07	0.10	7.92
**P**	0.06	0.04	0.26	0.59	0.55	1.39	0.49	0.06
**Mg**	0.26	0.26	0.13	0.36	0.34	0.84	0.18	0.18
**K**	1.02	1.70	0.39	2.59	2.57	1.58	1.08	0.24
**S**	0.09	0.13	0.1	0.38	0.35	0.17	0.16	2.79
**Na**	0.30	0.15	0.04	0.06	0.05	0.20	0.05	0.30
**Cl**	0.46	0.88	0.06	0.02	0.02	0.09	0.07	1.43
**Fe, mg/kg of DM**	973	782	25.0	116	143	199	127	5003
**Mn, mg/kg of DM**	347	107	7.00	41.0	56.0	160	120	4915
**Zn, mg/kg of DM**	19.0	7.00	16.0	54.0	55.0	50.0	113	9743
**Cu, mg/kg of DM**	3.00	2.00	2.00	16.0	16.0	10.0	14.0	989

a SBM = soybean meal.

b Minerals & vitamins was a commercial premix of minerals and vitamins supplied by Jiangsu AgriPortal Co. Ltd (Nanjing, Jiangsu).

c SP = soluble protein.

**Table 2:** Ingredient and nutrient composition of experimental diets.

Item	Diet (% RDP)			
	11.0	10.0	9.0	8.0
**Ingredient, % of DM**				
** Rice straw**	36.2	36.2	36.2	36.2
** Wheat straw**	12.1	12.1	12.1	12.1
** Coarsely cracked corn**	26.2	29.0	31.8	34.6
** Solvent soybean meal**	15.7	10.5	5.23	-
** Extruded soybean meal**	-	2.82	5.63	8.45
** Rice bran**	4.00	4.00	4.00	4.00
** Wheat middlings**	1.21	0.80	0.40	-
** Premix of mineral & vitamins**	3.75	3.75	3.75	3.75
** Urea**	0.85	0.85	0.80	0.84
**Nutrient composition, % DM**				
** DM, % of fresh matter**	88.9	89.0	89.0	89.0
** CP, % of DM**	17.2	15.9	14.6	13.3
** Soluble protein, % of DM**	5.79	5.25	4.72	4.18
** RDP^a^, % of DM**	10.9	10.0	9.09	8.09
** RUP^b^, % of DM**	6.25	5.85	5.52	5.25
** NDF, % of DM**	40.5	40.5	40.5	40.6
** ADF, % of DM**	26.0	25.8	25.6	25.4
** ADL, % of DM**	3.01	3.02	3.03	3.03
** Starch, % of DM**	23.3	25.1	26.8	28.6
** WSC, % of DM**	7.65	7.17	6.68	6.20
** Ether extract, % of DM**	2.88	2.89	2.91	2.93
** Ash, % of DM**	10.1	10.0	9.86	9.72
** MEAG^c^,^d^, g/d**	358	346	334	313
** ME^d^, % of requirement**	170	177	181	185
** MPAG^d^,^e^, g/d**	363	387	392	401
** MP^d^, % of requirement**	179	175	174	169

a RDP was the estimated value according to NASEM (2021).

b RUP was the estimated value according to NASEM (2021).

c MEAG = metabolizable energy-allowable gain.

d Estimated based on NRC (2007) using actual DMI, ADG, and analyzed nutrient composition of ingredients for this experiment.

e MPAG = metabolizable protein-allowable gain.

### Growth performance and rumen fermentation

The effect of dietary RDP concentration on growth performance, income over feed cost, and body measurements is shown in Table 3. Initial BW was similar among treatments and averaged 29.3 kg, and similarly, final BW and ADG were not impacted by treatment. Treatment had no effects on DMI (mean = 1.84 kg/d) and feed conversion ratio (**FCR**, mean = 7.59 kg/kg). Income over feed cost was not modified by dietary RDP concentration; however, it was numerically highest and increased by 20% for 11.1% RDP diet versus other lower RDP diets. Moreover, lowering dietary RDP content linearly decreased (*P* *=* 0.017) body height and tended (*P* *=* 0.079) to decrease chest depth. There was (*P* *=* 0.008) a quadratic effect of dietary RDP level for chest width, with maximal value at 9.0% RDP. Dietary RDP level did not impact other body measurements including withers height, rump height, body length, and chest girth.

**Table 3:** The effect of dietary RDP level on growth performance, income over feed cost, and body measurements in growing Hu sheep.

Item	Diet (% RDP)				SEM	*P*-value		
	11.0	10.0	9.0	8.0		Linear	Quadratic	Cubic
**Initial BW, kg**	29.6	29.3	29.4	28.9	1.02	0.167	0.617	0.476
**Final BW, kg**	45.8	45.0	44.8	44.0	1.11	0.136	0.978	0.795
**ADG, g/d**	262	253	248	245	13.0	0.338	0.813	0.963
**DMI, kg/d**	1.82	1.85	1.85	1.82	0.03	0.935	0.336	0.978
**FCR^1^, kg/kg**	7.19	7.55	7.70	7.91	0.38	0.175	0.846	0.871
**IOFC^2^, RMB**	101	88.7	82.1	83.4	15.2	0.378	0.648	0.979
**Body measurement indices, cm**								
** Body height**	72.0	71.0	69.8	69.5	0.78	0.017	0.686	0.776
** Withers height**	69.5	67.8	66.8	67.3	1.12	0.139	0.355	0.871
** Rump height**	71.7	70.9	70.6	69.9	0.95	0.145	0.977	0.787
** Body length**	75.7	76.6	75.1	75.5	0.97	0.574	0.771	0.302
** Chest depth**	34.9	34.0	34.3	33.1	0.70	0.079	0.832	0.362
** Chest width**	24.1^b^	25.0^ab^	26.1^a^	24.3^ab^	0.05	0.460	0.008	0.167
** Chest girth**	82.0	83.6	83.1	82.6	0.99	0.784	0.227	0.585

a,b Within a row, means without a common superscript letter differ (*P* *≤* 0.05).

1 FCR = DMI ÷ ADG.

2 IOFC = income over feed cost.

The effect of dietary RDP content on rumen fermentation is presented in Table 4. Treatment had no effect on ruminal pH. Concentrations of branched-chain VFA (i.e., isobutyrate, valerate, and isovalerate) in ruminal fluid decreased (*P* *≤* 0.047) linearly with decreasing dietary RDP level. There were (*P* *≤* 0.018) quadratic effects of RDP content on ruminal concentrations of total VFA, acetate, butyrate, and branched-chain VFA, with highest values at 10.0% RDP diet. Ruminal propionate concentration tended (*P* *=* 0.093) to respond quadratically to decreasing RDP. The ratio of acetate to propionate averaged 1.51 and was not different by dietary RDP content. The molar proportions of acetate, propionate, and butyrate did not change when dietary RDP level decreased from 11.0% to 8.0% of ration DM; however, those of branched-chain VFA decreased (*P* *≤* 0.002) linearly in response to decreasing RDP concentration. When decreasing RDP, the molar proportion of isobutyrate responded (*P* *=* 0.032) quadratically and that of butyrate tended (*P* *=* 0.080) to respond quadratically.

**Table 4:** The effect of dietary RDP level on ruminal fermentation in growing Hu sheep.

Item	Diet (%RDP)				SEM	*P*-value		
	11.0	10.0	9.0	8.0		Linear	Quadratic	Cubic
**pH**	6.55	6.51	6.32	6.46	0.07	0.130	0.184	0.110
**Total VFA, mM**	47.3	54.3	53.8	44.7	2.94	0.520	0.010	0.937
** Acetate, mM**	20.8	23.2	23.5	20.3	1.02	0.762	0.010	0.742
** Propionate, mM**	14.4	16.1	15.9	13.5	1.20	0.599	0.093	0.962
** Butyrate, mM**	9.70	12.2	11.9	9.28	0.96	0.711	0.013	0.894
** Isobutyrate, mM**	0.49^ab^	0.59^a^	0.50^ab^	0.31^b^	0.05	0.020	0.011	0.718
** Valerate, mM**	1.44^ab^	1.64^a^	1.53^ab^	1.08^b^	0.13	0.047	0.018	0.929
** Isovalerate, mM**	0.50^a^	0.59^a^	0.48^ab^	0.28^b^	0.06	0.004	0.015	0.613
** A:P ratio^1^**	1.52	1.50	1.54	1.66	0.13	0.435	0.610	0.991
**Molar proportions, %**								
** Acetate**	44.4	43.2	44.1	46.2	1.35	0.320	0.218	0.895
** Propionate**	30.5	29.6	29.4	29.5	1.53	0.667	0.745	0.953
** Butyrate**	20.1	22.1	21.9	20.6	0.94	0.736	0.080	0.814
** Isobutyrate**	1.00^a^	1.08^a^	0.93^ab^	0.69^b^	0.07	0.002	0.032	0.629
** Valerate**	2.98^a^	2.96^a^	2.80^ab^	2.37^b^	0.13	0.000	0.100	0.840
** Isovalerate**	1.03^a^	1.08^a^	0.87^ab^	0.60^b^	0.08	0.000	0.004	0.554

a,b Within a row, means without a common superscript letter differ (*P* *≤* 0.05).

1 A:P ratio = the ratio of acetate to propionate.

### Nutrient digestibility and N utilization

The effect of dietary RDP content on nutrient intake and apparent total-tract digestibility of nutrients is reported in Table 5. Intakes of CP and starch decreased (*P* *<* 0.001) and increased (*P* *=* 0.007) in a linear manner, respectively, as dietary RDP concentration decreased. Intakes of DM, ADF, and ether extract responded (*P* *≤* 0.049) cubically, and those of CP, NDF, and starch tended (*P* *≥* 0.051) to respond cubically, with decreasing dietary RDP. Furthermore, apparent total-tract digestibilities of CP and ADF decreased (*P* *≤* 0.001) in a linear manner and that of starch increased (*P* *=* 0.003) linearly, as dietary RDP content reduced. A trend (*P* *=* 0.051) for a quadratic effect was observed for NDF ­digestibility, as dietary RDP decreased. Apparent total-tract digestibilities of CP and starch tended (*P* *≤* 0.093) to respond cubically to decreasing RDP.

**Table 5:** The effect of RDP level on nutrient intake and apparent total-tract digestibility in growing Hu sheep.

Item	Diet (%RDP)				SEM	*P*-value		
	11.0	10.0	9.0	8.0		Linear	Quadratic	Cubic
**Nutrient intake, kg/d**								
** DM**	1.84	1.72	1.88	1.69	0.07	0.324	0.567	0.048
** OM**	1.78	1.68	1.80	1.66	0.07	0.412	0.735	0.115
** CP**	0.33^a^	0.29^b^	0.29^ab^	0.24^c^	0.01	0.000	0.694	0.051
** NDF**	0.84	0.79	0.86	0.77	0.03	0.327	0.597	0.054
** ADF**	0.54	0.50	0.54	0.48	0.02	0.167	0.595	0.049
** Starch**	0.48^b^	0.49^b^	0.57^a^	0.54^ab^	0.02	0.007	0.461	0.052
** Ether extract**	0.06	0.05	0.06	0.06	0.00	0.849	0.671	0.028
**Apparent total-tract digestibility, %**								
** DM**	57.9	62.0	56.6	57.0	1.67	0.429	0.675	0.240
** OM**	62.4	65.9	60.1	61.6	1.54	0.353	0.943	0.111
** CP**	77.0^a^	76.6^a^	68.8^b^	67.7^b^	1.32	0.000	0.697	0.093
** NDF**	27.5	34.6	29.4	22.2	2.79	0.154	0.051	0.980
** ADF**	24.0^a^	29.7^a^	19.6^ab^	15.7^b^	2.61	0.001	0.182	0.240
** Starch**	88.0^b^	89.5^a^	88.6^ab^	90.1^a^	0.47	0.003	0.640	0.063
** Ether extract**	50.8	52.8	49.3	47.9	2.23	0.208	0.449	0.460

a,b ^**,c**^Within a row, means without a common superscript letter differ (*P* *≤* 0.05).

The impact of RDP level on urinary N metabolites and N ­utilization is presented in Table 6. There were (*P* *≤* 0.001) linear decreases for estimated urine volume (from 1.68 to 0.87 L/d), urinary output of urea-N (from 18.4 to 9.91 g/d) and total-N (from 23.7 to 13.2 g/d), and the ratio of urinary N output to N intake (from 46.2% to 34.0%), as dietary RDP decreased from 11.0% to 8.0%. Urinary creatinine concentration and fecal N-to-N intake increased (*P* *≤* 0.004) linearly from 438 and 753 mg/L and 25.1% to 32.7%, respectively, in respond to reducing RDP. Moreover, lowering RDP resulted (*P* *≤* 0.003) in quadratic decreases in estimated urine volume, urinary total-N, and urinary N to N intake. There were (*P* *=* 0.016) quadratic effects of dietary RDP concentration on retained N and the ratio of retained N to N intake, with maximal values at 10.0% RDP. Lowering RDP tended (*P* *≤* 0.092) to cause a quadratic increase in urinary creatinine content and a quadratic decrease in urinary urea-N output. Fecal total N responded (*P* *=* 0.027) cubically and fecal N/N intake and retained N/N intake tended (*P* *≤* 0.098) to respond cubically to decreasing RDP.

**Table 6:** The effect of RDP level on urinary N metabolites and N utilization in growing Hu sheep.

Items	Diet (%RDP)				SEM	*P*-value		
	11.0	10.0	9.0	8.0		Linear	Quadratic	Cubic
**Urinary creatinine content, mg/L**	438^a^	638^ab^	840^a^	753^a^	85.4	0.004	0.092	0.348
**Estimated urine volume^1^, L/d**	1.68^a^	1.23^ab^	0.87^b^	1.03^b^	0.14	0.001	0.030	0.382
**Urinary urea-N output, g/d**	18.4^a^	13.1^b^	12.1^bc^	9.91^c^	0.68	0.000	0.060	0.146
**Urinary total-N output, g/d**	23.7^a^	16.9^b^	15.2^bc^	13.2^c^	0.80	0.000	0.001	0.175
**Fecal total-N output, g/d**	12.9	11.0	13.4	12.3	0.76	0.836	0.596	0.027
**Retained-N, g/d**	14.8^ab^	21.7^a^	16.6^ab^	13.1^b^	1.87	0.179	0.013	0.110
**Urinary N/N intake, %**	46.2^a^	34.7^b^	34.0^b^	34.9^b^	2.06	0.001	0.003	0.120
**Fecal N/N intake, %**	25.1^ab^	22.4^b^	30.1^ab^	32.7^a^	1.93	0.001	0.202	0.081
**Retained N/N intake, %**	29.5^b^	43.4^a^	35.8^ab^	32.5^ab^	3.28	0.928	0.016	0.098

a–c Within a row, means without a common superscript letter differ (*P* *≤* 0.05).

1 Estimated assuming a constant creatinine excretion rate of 15.0 mg/kg of BW (Pereira et al. 2022).

## Discussion

In recent years, pelleted TMR has been commonly used in the Chinese sheep sector, especially for smaller farmers. Compared with conventional TMR, pelleted TMR has been proved to prevent feed sorting and improve growth performance in sheep (Zhong et al. 2018; Zhang et al. 2019; Malik et al. 2021). In our study, all the animals were fed pelleted TMR produced and delivered by a local feed company. Based on NRC (2007), NASEM (2021), the mean RDP content of coarsely cracked corn, extruded soybean meal, solvent soybean meal, and wheat middlings are 41.7%, 64.8%, 60.4%, and 90.7% of CP, respectively, thus, estimated RDP concentrations (% of DM) of these ingredients herein were 3.50%, 31.6%, 29.3%, 17.6%, respectively. The substitution of solvent-extracted soybean meal and wheat middlings with coarsely cracked corn and extruded soybean meal were able to achieve dietary RDP levels of 10.2%, 9.43%, 8.63%, and 7.83%, respectively. In our study, NASEM (2021)-estimated RDP content, measured using an in situ method, was 10.9%, 10.0%, 9.09%, and 8.09%, respectively. NASEM (2021)-derived RDP content was similar to those reported by NRC (2007) and, more importantly, reflected more recent data. Consequently, NASEM (2021)-derived RDP concentrations were used in our study herein. Reynal and Broderick (2005) substituted solvent soybean meal and urea with lignosulfonate-treated soybean meal in order to decrease dietary RDP from 13.2% to 10.6% of ration DM (i.e., difference = −2.6% units) with similar RUP concentrations (mean = 6.2% of ration DM, difference = +0.8% unit) in lactating dairy cows. Kalscheur et al. (2006) replaced soybean meal with ground corn and nonenzymatically browned soybean meal to reduce dietary RDP level from 11.0% to 6.8% in a study with lactating cows.

Our study did not observe any differences in final BW or ADG among varying RDP levels. Contrary to our results, Wang et al. (2020) investigated the impact of dietary CP level (i.e., 14.1%, 12.1%, and 10.1% of ration DM) in castrated Tibetan sheep and reported that final BW during d 36 to 70 and d 71 to 105 were higher for 14.1% and 12.1% CP as compared with 10.1% CP; whereas it was not impacted by treatment for d 1 to 35. Dabiri and Thonney (2004) conducted two sheep trials feeding 3 levels of dietary CP (i.e., 17.0%, 15.0%, and 13.0%) to lambs for 42 d and observed that average daily gain showed linear decreases from 312 to 267 g/d and from 317 to 279 g/d, respectively as dietary CP decreased in the two trials. Ross et al. (2024) fed 3 levels of RDP (i.e., 10.0%, 9.0%, and 8.0%; 17.4%, 16.4%, and 15.4% CP, respectively) to finishing beef steers and stated that final BW did not change during the whole 139-d experiment but ADG decreased linearly during first 35 d. Responses of final BW and ADG to varying RDP may be dependent on the effects of RDP on microbial protein synthesis and ruminal carbohydrate fermentation, thereby impacting dietary supplies of MP and ME (Firkins 1996; Owens et al. 2014). In agreement with our results, DMI was not modified by dietary CP and RDP level, ranging from approximately 17.0% to 13.0% and 10.0% to 8.0%, respectively (Dabiri and Thonney 2004; Saro et al. 2020; Ross et al. 2024), suggesting that dietary CP or RDP has limited effect on feed intake in growing sheep and beef steers.

There were quadratic decreases of ruminal concentrations of total VFA, acetate, propionate, butyrate, and branched-chain VFA to reducing RDP level in the current study (i.e., the maximal values observed at 10.0% RDP). Earlier, Reynal and Broderick (2005) stated that ruminal total VFA and propionate concentrations showed quadratic increases with reducing dietary RDP level from 13.2% to 10.6% and reached their minimal values at 12.3% RDP. However, ruminal total VFA concentration did not change when feeding 11.5% versus 9.3% RDP (Sun et al. 2019), and concentrations of total VFA and most of individual VFA were not different between 11.6% and 9.4% RDP diets (Hristov et al. 2004). These various responses to the changes in dietary RDP concentration may be related to diet composition, sampling time, and physiological phase, etc. In our study, lowering RDP led to linear decreases in ruminal concentrations and molar proportions of individual branched-chain VFA. Given that branched-chain VFA are formed by the deamination and decarboxylation of branched-chain AA (Allison 1970), the decline in branched-chain VFA may be partly attributed to reduced supply of protein and branched-chain AA, as supported by Zhang et al. (2013), in which total branched-chain VFA linearly decreased as the supplemental amount of branched-chain AA reduced. The molar proportions of acetate, propionate, and butyrate in our study averaged 44.5%, 29.8%, and 21.2%, respectively. Based on a meta-analysis by Noziere et al. (2011), the molar percentages of these VFA in the rumen fluid ranged from 39.3% to 79.1%, 11.0% to 47.0%, and 4.4% to 20.0%, respectively. The large proportion of butyrate in the rumen observed herein may be partially explained by high WSC concentration (i.e., mean = 6.9% of ration DM). Earlier studies have demonstrated that elevated supply of sugars such as lactose and malate can lead to enhanced ruminal butyrate synthesis (Martin et al. 2000; DeFrain et al. 2004).

Similar to our study, the declines in dietary CP content from 16.0% to 10.2% or from 17.6% to 13.3% did not impact DM and OM digestibility (Kaya et al. 2009; Prima et al. 2019). We observed that apparent total-tract digestibilities of CP and ADF decreased linearly in response to decreasing RDP level. Consistent with our results, Lee et al. (2012) stated that apparent total-tract digestibilities of ADF and CP were lower for a MP-adequate diet (15.7% CP with 9.8% RDP, and 5.9% RUP) versus a MP-deficient diet (13.6% CP with 9.1% RDP and 4.4% RUP). Similar patterns were also reported with feeding 16.7% CP (i.e., 10.6% RDP and 6.1% RUP) versus 14.8% CP (9.8% RDP and 4.9% RUP) in lactating dairy cows (Lee et al. 2011). Soto et al. (1994) demonstrated that supplementation of peptides and AA stimulated the growth of cellulolytic bacteria in pure cultures. Subsequently, Reis et al. (2016) found that casein-based RDP supplementation enhanced fiber degradation but did not impact bacterial profile in the rumen. Our study revealed remarkably low apparent total-tract digestibilities of NDF and ADF, with averages of 28.4% and 22.3%, respectively. It may be due to the fact that the use of lignin as an internal marker can underestimate fiber digestibility due to incomplete fecal recovery of lignin (Kanani et al. 2014).

In the current study, when reducing dietary RDP concentration, estimated urine volume and urinary urea-nitrogen and total-nitrogen output showed linear and quadratic decreases. Similar with our findings, urine volume, urinary urea-nitrogen, and urinary total-nitrogen excretion decreased linearly when feeding decreasing CP content ranging from 19.4% to 13.5% (Broderick 2003; Olmos Colmenero and Broderick 2006). Based on a meta-analysis with lactating dairy cows, urinary urea-nitrogen excretion can be best predicted by the combination of milk urea-nitrogen and dietary CP concentration, while urinary nitrogen excretion can be best estimated through the combination of milk urea nitrogen, dietary CP level, and DMI (Spek et al. 2013). The amount of RDP or CP exceed the requirements is absorbed by the rumen wall as ammonia, and ammonia is then converted into urea in urine (Bach et al. 2005); elevated excretion of nitrogen metabolites may require more water to dilute these metabolites and thus increases urine volume (Valadares et al. 1999; Broderick 2003; Olmos Colmenero and Broderick 2006). Although fecal nitrogen output responded cubically, it changed slightly (difference = 2.4 g/d). Reynal and Broderick (2005) reported that decreasing dietary RDP from 13.2% to 10.6% did not alter fecal nitrogen output in dairy cows. Hence, it appears that nitrogen consumed in excess of the requirements is primarily excreted via urination. In the current study, nitrogen retention and the ratio of retained nitrogen to nitrogen intake showed quadratic responses and reached their maximal values at 10.0% RDP. There is an apparent pattern for nitrogen retention to increase when increasing dietary CP content (Al Jassim et al. 1991; Prima et al. 2019). The numerically lower N retention with feeding 11.0% vs. 10.0% RDP may be partly derived from estimated error of urine volume and urinary total nitrogen. On the other hand, excessive RDP supply with 11.0% RDP diet may require more energy to excrete urea via urine and thus may lead to less energy to support growth in Hu sheep. The quadratic maxima observed at 10.0% RDP indicated that the efficiency of nitrogen utilization (i.e., retained N/N intake) requires adequate but not excess RDP supply to maximize microbial protein synthesis in the rumen. It is worth mentioning that our study observed the ratio of retained nitrogen to nitrogen intake between 29.5% and 43.4%, a range greater than the 26.2% to 36.2% reported by Seoni et al. (2021) studying lambs with an average BW of 24.5 kg. The overestimation may have resulted from the use of creatinine and lignin for the estimation of urine volume and fecal output, respectively.

## Conclusions

Contrary to our hypothesis, rumen fermentation, fiber digestibility, and the efficiency of N utilization (i.e., nitrogen retained/nitrogen intake) were optimized when feeding the 10.0% RDP diet. Reducing dietary RDP content from 11.0% to 8.0% ration DM linearly decreased urinary nitrogen excretion but did not negatively impact growth performance in Hu sheep. While no statistical significance was found, feeding the 11.0% RDP diet led to the greatest income over feed cost (101 vs. 84.7 RMB) when compared to lower RDP concentrations. Therefore, incorporating economic considerations is crucial for future investigations on low-protein diets for sheep.

## Abbreviations:

AA: amino acids
ADF: acid detergent fiber
ADG: average daily gain
ADICP: acid detergent insoluble crude protein
ADL: acid detergent lignin
BW: body weight
CP: crude protein
DM: dry matter
DMI: dry matter intake
FCR: feed conversion ratio
ME: metabolizable energy
MP: metabolizable protein
N: nitrogen
NDF: neutral detergent fiber
NDICP: neutral detergent insoluble crude protein
RDP: rumen degradable protein
RUP: rumen undegradable protein
TMR: total mixed ration
VFA: volatile fatty acids
WSC: water soluble carbohydrates
